# Supplementary figures and images for: Basal–epithelial subpopulations underlie and predict chemotherapy resistance in triple-negative breast cancer
Source: EMBO Mol Med. 2024 Mar 13;16(4):823–53. doi: 10.1038/s44321-024-00050-0 (PMC11018633; doi:10.1038/s44321-024-00050-0)

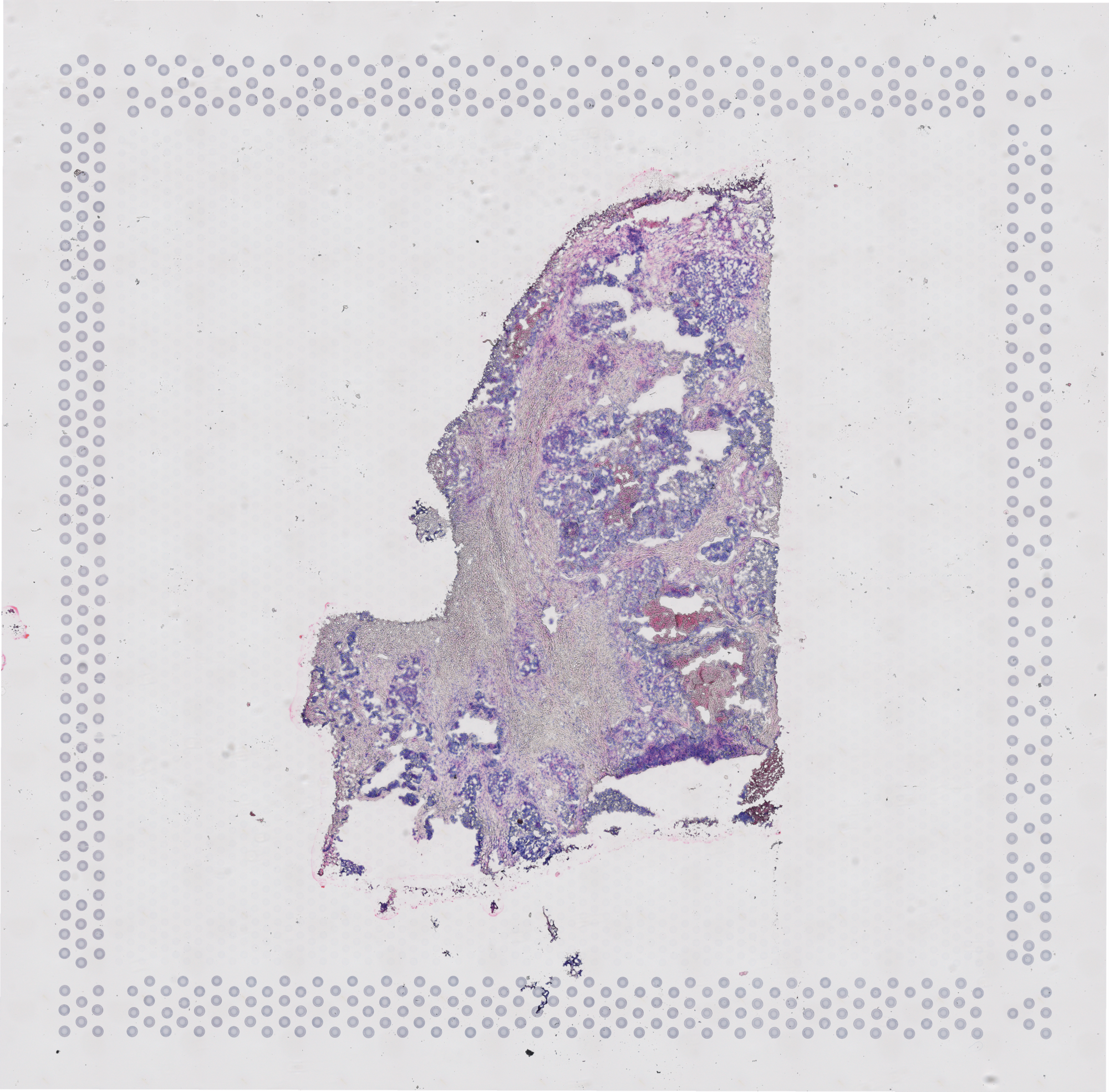

Supplement: Supplementary file 3 — Source Data Fig. 1 [file 44321_2024_50_MOESM3_ESM.zip › 1F Image data.png]

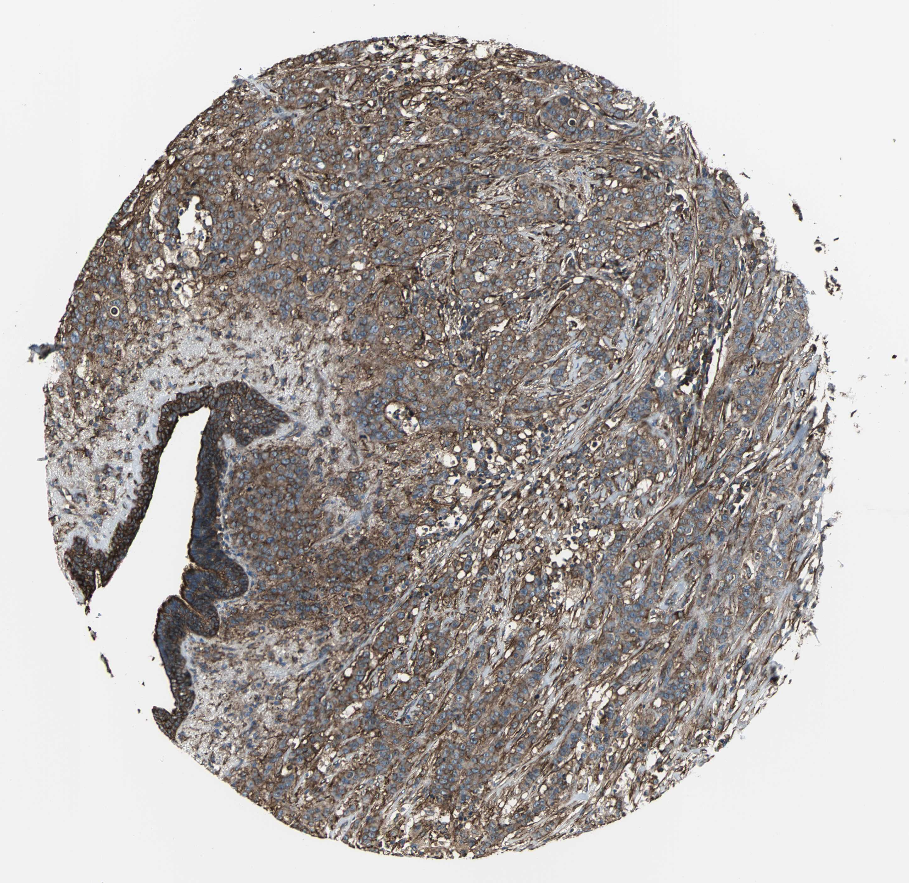

Supplement: Supplementary file 10 — Source Data Fig. 8 [file 44321_2024_50_MOESM10_ESM.zip › 8E_breast_cancer.png]

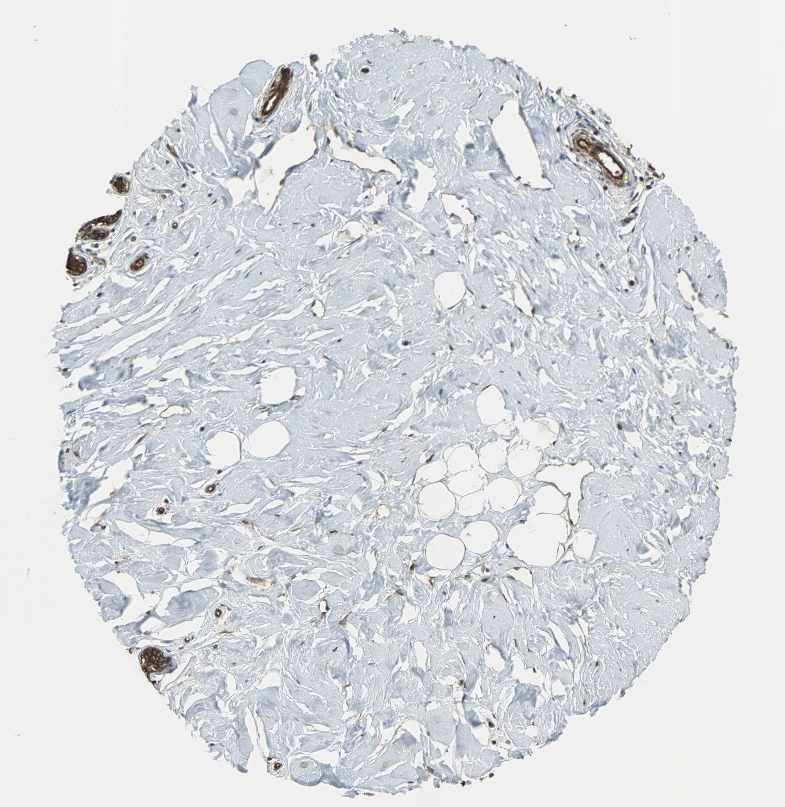

Supplement: Supplementary file 10 — Source Data Fig. 8 [file 44321_2024_50_MOESM10_ESM.zip › 8E_normal_breast.png]
