# Supplementary material for: Basal–epithelial subpopulations underlie and predict chemotherapy resistance in triple-negative breast cancer
Source: EMBO Mol Med. 2024 Mar 13;16(4):823–53. doi: 10.1038/s44321-024-00050-0 (PMC11018633; doi:10.1038/s44321-024-00050-0)
Supplement: Supplementary file 11 — Expanded View Figures [file 44321_2024_50_MOESM11_ESM.pdf]

Expanded View Figures

Figure EV1. (**A**) scRNA-seq data analysis of pre-treated primary TNBC patients identified a similar subpopulation in two independent scRNA-seq of TNBC datasets. The cell clusters marked under lines are representing, cells belonging to the breast cancer subtype. The genes defining each cluster were annotated against the cellmarker database and cell-type identities were assigned to each cluster. (**B**) Expression of metastasis-associated genes in pre-treated TNBC patients scRNA-seq datasets. The metastasis signature of 49 genes was used by Lawson et al, 2015 and their average expression was plotted across each cluster in both datasets. (**C**) Chemoresistance signature of 143 genes was used by Balko et al, 2012 and their average expression was plotted across each cell types in both datasets. (**B**, **C**) The significance test of expression levels of metastasis and chemoresistance genes between the cell types was performed using two-tailed unpaired Wilcoxon test in stat_compare_means() of ggpubr package. (**D**) The violin plots show expression of malignant cell markers of Luminal and basal breast cancer type. The cancer cell marker of basal and luminal epithelial type was retrieved from CellMarker database and plotted on our primary TNBC dataset. (**E**) Spatial transcriptome data of two recurrent TNBC patients. The left plots show the H&E staining (scale bar, 10 µm) of two TNBC tumors. The middle plot shows the spatial location of basal epithelial cells within these spatial datasets. Right plot showing the mean expression of our 101 signature genes in these spatial transcriptome datasets.

▶

 © The Author(s)

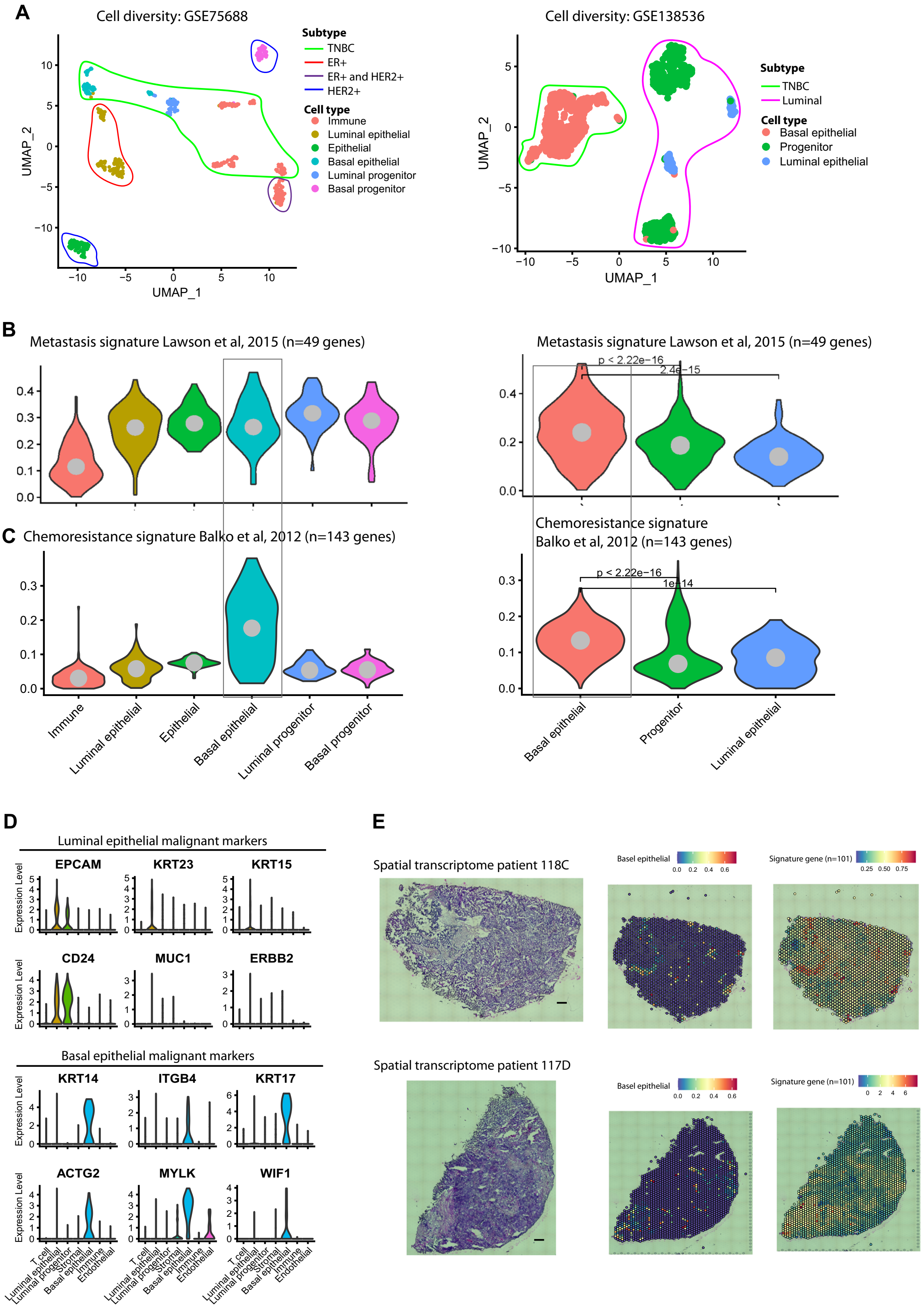

A
Cell diversity: GSE75688
Subtype
TNBC
ER+
ER+ and HER2+
HER2+
Cell type
Immune
Luminal epithelial
Epithelial
Basal epithelial
Luminal progenitor
Basal progenitor
UMAP_1
UMAP_2
Cell diversity: GSE138536
Subtype
TNBC
Luminal
Cell type
Basal epithelial
Progenitor
Luminal epithelial
B
Metastasis signature Lawson et al, 2015 (n=49 genes)
p < 2.22e−16
2.4e−15
C
Chemoresistance signature Balko et al, 2012 (n=143 genes)
Chemoresistance signature
Balko et al, 2012 (n=143 genes)
p < 2.22e−16
1e−14
Immune
Luminal epithelial
Epithelial
Basal epithelial
Luminal progenitor
Basal progenitor
Progenitor
D
Luminal epithelial malignant markers
EPCAM
KRT23
KRT15
CD24
MUC1
ERBB2
Basal epithelial malignant markers
KRT14
ITGB4
KRT17
ACTG2
MYLK
WIF1
Expression Level
T cell
Luminal epithelial
Luminal progenitor
Stromal
Basal epithelial
Immune
Endothelial
E
Spatial transcriptome patient 118C
Basel epithelial
Signature gene (n=101)
Spatial transcriptome patient 117D

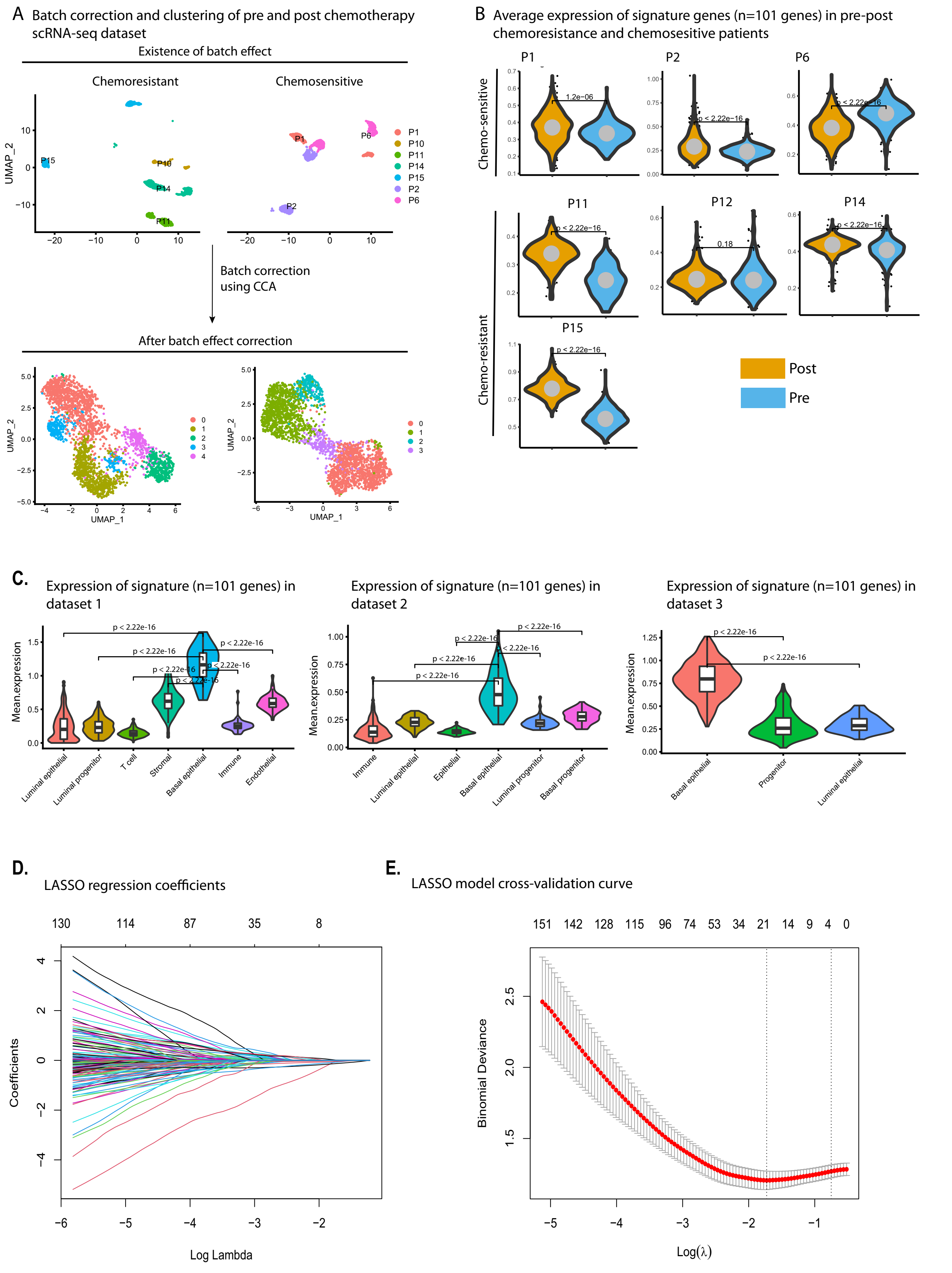
A Batch correction and clustering of pre and post chemotherapy scRNA-seq dataset
Existence of batch effect
Chemoresistant
Chemosensitive
UMAP_2
P1
P10
P11
P14
P15
P2
P6
Batch correction using CCA
After batch effect correction
UMAP_1
B Average expression of signature genes (n=101 genes) in pre-post chemoresistance and chemosesitive patients
Chemo-sensitive
Chemo-resistant
1.2e−06
p < 2.22e−16
0.18
Post
Pre
C. Expression of signature (n=101 genes) in dataset 1
Expression of signature (n=101 genes) in dataset 2
Expression of signature (n=101 genes) in dataset 3
Mean.expression
Luminal epithelial
Luminal progenitor
T cell
Stromal
Basal epithelial
Immune
Endothelial
Epithelial
Basal progenitor
Progenitor
D. LASSO regression coefficients
Coefficients
Log Lambda
E. LASSO model cross-validation curve
Binomial Deviance
Log(λ)

 © The Author(s)

◀ **Figure EV2.** (**A**) The upper umap plot shows existence of possible batch effect in resistant and sensitive datasets. The batch effects regress out using canonical correlation analysis (CCA) and samples were integrated. The bottom umap plots shows removal of possible batch effects from the datasets. Total cluster identified in the single-cell datasets of 7 TNBC patients pre- and post chemotherapy are also shown in the same bottom plot. (**B**) The average expression profile of signature genes in each patient of pre- and post-treated groups. The significance testing of expression of signature genes between the chemotherapy-treated and untreated groups was performed using two-tailed unpaired Wilcoxon test in stat_compare_means() of ggpubr package. (**C**) Violin plot showing average expression of signature genes across clusters of all three primary TNBC tumor datasets. The average expression of all 101 signature genes was plotted in all three primary TNBC scRNA-seq datasets and confirmed their activation in similar subpopulations of basal epithelial cells. In the box-and-whisker within violin plots, the horizontal lines mark the median, the box limits indicate the 25th and 75th percentiles, and the whiskers extend to 1.5× the interquartile range from the 25th and 75th percentiles. The significance test of expression levels of signature genes between the cell types was performed using two-tailed unpaired Wilcoxon test in stat_compare_means() of ggpubr package. (**D**) The coefficients from the Lasso fit represent the contributions of the 20 genes expression in the model. The plot shows lasso regression coefficient values in which each curve corresponds to a variable. It shows the path of its coefficient against the Log Lamda of the whole coefficient vector as λ varies. The axis above indicates the number of nonzero coefficients at the current λ, which is the effective degrees of freedom (df) for the lasso. (**E**) The selection of tuning parameter (λ) in the LASSO model based on the tenfold cross-validation. The plots are showing a cross-validation curve (red dotted line) along with mean binomial deviance against a range of Log(λ). The vertical dotted lines represent lambda.min and lambda.1se. This panel shows the changes in partial likelihood deviance with λ values. The 20 genes were selected according to the most regularized model such that the error is within one standard error of the minimum.

© The Author(s)

A. Ligand-receptor activity in LAMININ

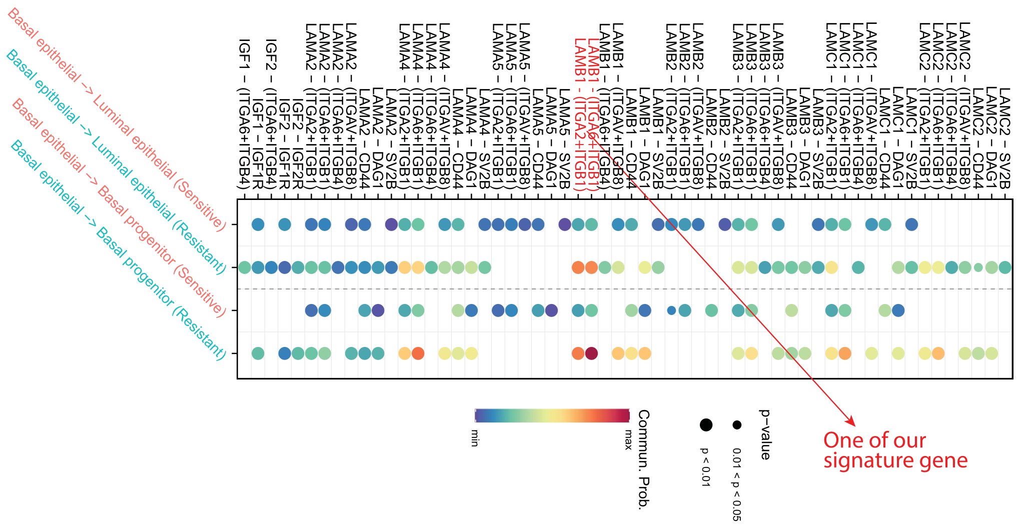

B. Candidate gene matrix

Gene	Chemoresistance: scRNA-seq	Chemoresistance: Bulk RNA-seq	BC subtype: BC Tumors	BC subtype: BC cell lines	EMT: EMT-High TNBC (Tumors)	EMT: Mammary EMT (HMLE)	Survival (RFS): Poor survival (TNBC)	Survival (RFS): Poor survival (LN+ TNBC)
ACTG2	+	+	+	+	+	+	+	+
ACTN1	+	+	+	+	+	+	+	+
MYLK	+	+	+	+	+	+	+	+
ANXA1	+	+	+	+	+	+	+	-
CNN3	+	+	+	+	+	+	+	-
CAV2	NA	+	+	+	+	+	+	+
MSRB3	NA	+	+	+	+	+	+	+
SFRP1	+	+	+	+	+	+	-	+
TNC	NA	+	+	+	+	+	+	+
KRT17	NA	+	+	+	+	+	+	-
ADAMTS1	NA	+	+	+	+	+	-	+
FLNA	NA	+	+	+	+	+	-	+

C. ACTN1 expression across cancer cell lines (CCLE)

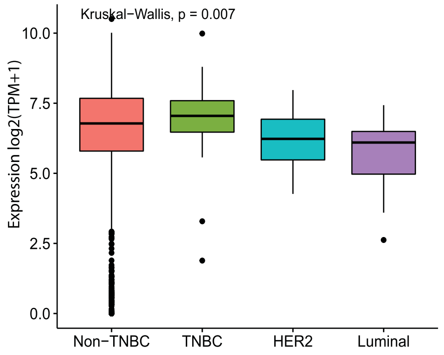

D. Expression of 20 gene signature across integrated dataset

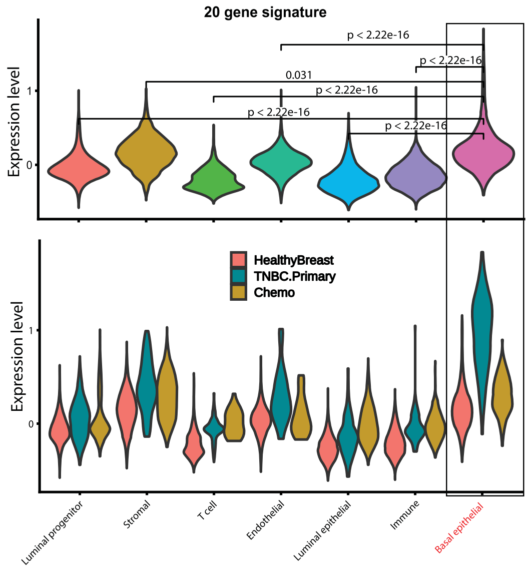

E. Expression of basal and luminal markers across integrated dataset

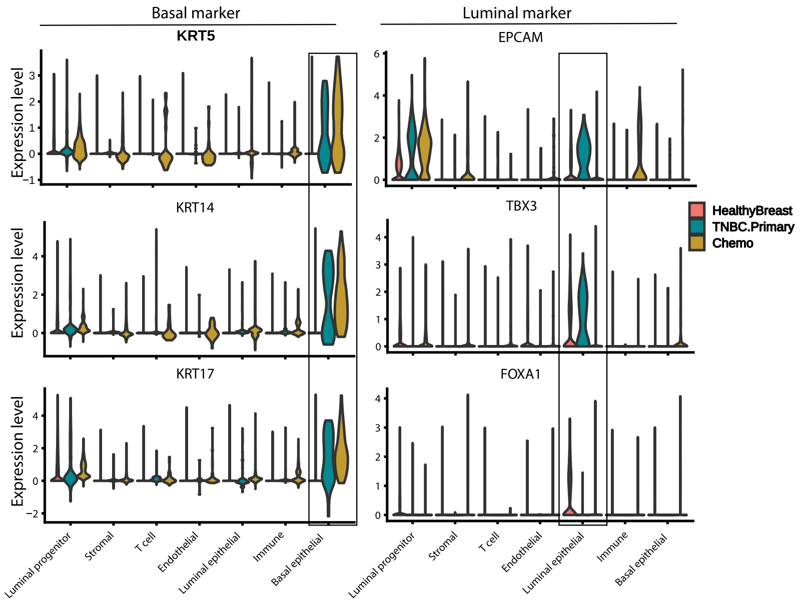

 © The Author(s)

◀ **Figure EV3.** (**A**) Bar plot showing ligand receptor involved in intercellular signaling of LAMININ signaling pathway. The significance test between signaling pathway signals was computed using two-tailed unpaired Wilcoxon test using the presto package. (**B**) Table showing candidate gene ranking matrix. The "+" symbol indicates the presence and the "–" sign indicates the absence of a parameter in each of the genes. (**C**) Expression of *ACTN1* in TNBC, HER2, Luminal, and non-TNBC cell lines. Expression values were obtained from CCLE. The axis shows TNBC, HER2, Luminal, and non-TNBC cancer cell lines and the y axis is their mRNA expression levels. In the box-and-whisker plots, the horizontal lines mark the median, the box limits indicate the 25th and 75th percentiles, and the whiskers extend to 1.5× the interquartile range from the 25th and 75th percentiles. The significance test of expression levels of ACTN1 was performed using Kruskal–Wallis test in ggpubr package. (**D**) The expression of the 20-gene signature across the cell types shown in upper plot of healthy breast, primary TNBC and chemotherapy-treated TNBC data sets and lower plot shows dataset-wise expression profile. The statistical testing of expression levels of 20 gene was performed using two-tailed unpaired Wilcoxon test in stat_compare_means() of ggpubr package. (**E**) The expression of basal markers (left violin plots) and luminal epithelial (right violin plot) markers across cell types of the healthy breast, primary TNBC and chemotherapy-treated TNBC cells.

© The Author(s)
